# Safeguarding the Right to a Whole Life

**DOI:** 10.1002/cai2.70080

**Published:** 2026-08-18

**Authors:** Jie Qiao, Fei Ma

**Affiliations:** ^1^ State Key Laboratory of Female Fertility Promotion, Center for Reproductive Medicine Peking University Third Hospital Beijing China; ^2^ National Clinical Research Center for Obstetrics and Gynecology (Peking University Third Hospital) Beijing China; ^3^ Key Laboratory of Assisted Reproduction, Peking University Ministry of Education Beijing China; ^4^ Beijing Key Laboratory of Reproductive Endocrinology and Assisted Reproductive Technology Beijing China; ^5^ National Cancer Center/National Clinical Research Center for Cancer/Cancer Hospital Chinese Academy of Medical Sciences and Peking Union Medical College Beijing China

**Keywords:** cancer survivorship, fertility preservation, oncofertility

The ultimate concern of medicine transcends the mere eradication of disease; more profoundly, it resides in the preservation of human dignity and the intergenerational continuity of life's hope. From the ancient maxim of the *Zhouyi* (*Book of Changes*)—“The greatest virtue of Heaven and Earth is the giving of life”—to the contemporary advances in assisted reproductive technology that repeatedly render the impossible possible, the continuity and full flourishing of life have remained among medicine's deepest aspirations. As precision oncology continues to continually improve survival outcomes, we are increasingly cognizant that a truly curative path must not only draw patients back from the abyss of illness but also preserve their right to embrace a fulfilling life—of which safeguarding reproductive potential stands as an indispensable dimension that must not be overlooked [1].

With the remarkable advance in tumor diagnosis and treatment, a growing number of young patients have moved beyond the threshold of mere survival to reclaim the fullness of life. As long‐term survival becomes achievable for an increasing proportion of patients, a central consideration in redefining quality of life lies in the ability to “nurture the future” and to reclaim familial and societal identities. Taking breast cancer as an example, 50% of young patients have not yet completed their families at the time of diagnosis, yet the actual pregnancy rate remains below 5% [2, 3]. Cytotoxic chemotherapy may exert substantial gonadotoxic effects, whereas prolonged endocrine therapy may defer childbearing during the reproductive years; the reproductive effects of many targeted therapies remain incompletely characterized [4, 5]. Consequently, some patients who survive cancer may still face premature ovarian insufficiency and fertility loss. Reassuringly, existing evidence indicates that timely and appropriately selected interventions can preserve reproductive potential without sacrificing anticancer efficacy in many clinical settings, encompassing fertility preservation strategies like cryopreservation techniques and pharmacological protection [5, 6, 7]. Between oncology and reproductive medicine, an interdisciplinary frontier demanding systematic cultivation has thus emerged. Oncofertility stands as the nascent cross‐disciplinary practice that responds to this pressing clinical need [1]. We advocate a clinical philosophy wherein anticancer treatment and fertility preservation are considered in parallel, so that disease control and preservation of reproductive potential need not become mutually exclusive goals, thereby moving fertility preservation from an exploratory practice confined to selected centers toward routine clinical care, and from post‐treatment remediation toward proactive and safeguarding planning [7].

Since its inception, *Cancer Innovation* has steadfastly championed the examination of the full spectrum of oncologic diagnosis and treatment through an innovative perspective, emphasizing frontier strategies, interdisciplinary integration, and key enabling technologies. The journal has established a dedicated *“*Oncofertility*”* column, aiming to build a high‐quality interdisciplinary academic platform for scholars across oncology, reproductive medicine, gynecology, pediatrics, endocrinology, nursing science, psychology, medical ethics, and related disciplines. Through this initiative, we aspire to propel the optimization of clinical strategies, innovation in technical pathways, and the deepening of humanistic care—transforming fertility preservation from an optional consideration into a routine imperative within standardized oncologic practice, so that scientific rigor and humanistic concern may resonate in unison, and cancer control and preservation of reproductive potential may advance as concomitant pursuits.

Let us, in concert, inscribe a new chapter in fertility preservation for young patients with cancer in this new era, guided by enduring principles: the earlier, the better; care throughout the entire journey; survival and fertility—both within reach.

## Author Contributions

**Jie Qiao:** writing – original draft, writing – review and editing, conceptualization, investigation, project administration. **Fei Ma:** writing – original draft, writing – review and editing, conceptualization, investigation.

## Funding

The authors have nothing to report.

## Ethics Statement

The authors have nothing to report.

## Consent

The authors have nothing to report.

## Conflicts of Interest

Professor Fei Ma is a member of the *Cancer Innovation* Editorial Board. To minimize bias, he was excluded from all editorial decision‐making related to the acceptance of this article for publication. The other authors declare no conflicts of interest.

## Data Availability

Data sharing is not applicable to this article as no data sets were generated or analyzed during the current study.
